# Genomic Selection in Multi-environment Crop Trials

**DOI:** 10.1534/g3.116.027524

**Published:** 2016-03-11

**Authors:** Helena Oakey, Brian Cullis, Robin Thompson, Jordi Comadran, Claire Halpin, Robbie Waugh

**Affiliations:** * Division of Plant Sciences, University of Dundee at the James Hutton Institute, Invergowrie, Dundee DD2 5DA, Scotland, UK; † National Institute for Applied Statistics Research Australia, University of Wollongong, NSW, 2522, Australia; § Rothamsted Research, Harpenden, Hertfordshire AL5 3JQ, UK; ** Department of Cell and Molecular Sciences, The James Hutton Institute, Invergowrie, Dundee DD2 5DA, Scotland, UK

**Keywords:** multi-environment trial, genomic selection, random ridge regression, GEBV, barley, GenPred, shared data resource

## Abstract

Genomic selection in crop breeding introduces modeling challenges not found in animal studies. These include the need to accommodate replicate plants for each line, consider spatial variation in field trials, address line by environment interactions, and capture nonadditive effects. Here, we propose a flexible single-stage genomic selection approach that resolves these issues. Our linear mixed model incorporates spatial variation through environment-specific terms, and also randomization-based design terms. It considers marker, and marker by environment interactions using ridge regression best linear unbiased prediction to extend genomic selection to multiple environments. Since the approach uses the raw data from line replicates, the line genetic variation is partitioned into marker and nonmarker residual genetic variation (*i.e.*, additive and nonadditive effects). This results in a more precise estimate of marker genetic effects. Using barley height data from trials, in 2 different years, of up to 477 cultivars, we demonstrate that our new genomic selection model improves predictions compared to current models. Analyzing single trials revealed improvements in predictive ability of up to 5.7%. For the multiple environment trial (MET) model, combining both year trials improved predictive ability up to 11.4% compared to a single environment analysis. Benefits were significant even when fewer markers were used. Compared to a single-year standard model run with 3490 markers, our partitioned MET model achieved the same predictive ability using between 500 and 1000 markers depending on the trial. Our approach can be used to increase accuracy and confidence in the selection of the best lines for breeding and/or, to reduce costs by using fewer markers.

Whole genome prediction (WGP) uses genotypic information in the form of molecular genetic markers to predict individual phenotypic performance, and has utility in livestock and crop breeding. For a particular population, WGP associates a phenotypic value with each molecular marker allele, which is consequently known as a marker effect. The sum of the marker effects (which relate to the alleles present in an individual’s genotype) is a predictor of their phenotypic performance, and is known as a genomic estimated breeding value (GEBV). In ‘Genomic Selection’ (GS), the GEBV is used to select the best parents for breeding, or to predict the performance of progeny using only genotyping data without the need for phenotypic screening. In order to derive GEBVs for prediction, an initial ‘training population’ is phenotyped and genotyped to estimate marker effects. GEBVs can then be calculated for individuals descended from, or related to, the training population (a ‘validation population’) that have not been phenotyped, but for which genotypic information is available. This saves both time and the costs associated with phenotyping in a breeding program. Since GS uses all genetic markers to calculate the GEBV, it potentially captures all of the loci that influence a trait. This distinguishes GS from more traditional marker assisted selection (MAS), where a few diagnostic markers are used to follow the inheritance of specific loci influencing a trait, and marker assisted recurrent selection (MARS), where only a subset of significant markers are used to select for quantitative trait loci (QTL) in a given population. The potential of GS to accelerate crop improvement due to shorter generation times and the avoidance of phenotypic evaluation has been established (Jannink *et al.* 2010), and shown to outperform MAS (Heffner *et al.* 2010) and MARS (Massman *et al.* 2013).

Since 2001, when Meuwissen *et al.* (2001) compared least square, ridge regression best linear unbiased prediction (RR-BLUP) and two Bayesian approaches (BayesA and BayesB) for GS in animal breeding, there has been an increase in the number of methods available (Wang *et al.* 2012; de los Campos *et al.* 2013; Desta and Ortiz 2014), and widespread uptake, particularly in dairy cattle breeding, where official GEBVs are published (http://www.interbull.org). These GS methods, however, differ in their predictive ability (de los Campos *et al.* 2013) and suitability for specific applications. Crop populations may require different GS methods to those of animals due to the potential presence of extensive linkage disequilibrium (LD), population substructure, and agronomic performance traits which are often influenced by many QTL of small effect (Wimmer *et al.* 2013). In a simulated data set based on actual marker data from barley, RR-BLUP was found to be more accurate than BayesB (Zhong *et al.* 2009), and has been recommended for crop improvement applications (Heslot *et al.* 2012). Wimmer *et al.* (2013) compared the performance of four commonly used methods; RR-BLUP and BayesB (Meuwissen *et al.* 2001), LASSO (Tibshirani 1996), and the elastic net (Zou and Hastie 2005). They also found good performance with RR-BLUP, and recommended its use in crops. RR-BLUP has the advantage over Bayesian approaches of being easily implemented and quick.

Despite these advances, GS is only starting to be adopted in crop breeding. Some of the reluctance to adopt GS may be due to the additional modeling challenges of crop improvement scenarios. Suitable models need to accommodate data from replicate plants of the same line, the influence of spatial variation in the field, and potentially different ‘environments’ if the crop is trialed in several locations or multiple years. These variables introduce significant genotype by environment interactions (G × E) and new methods are needed that consider G × E effects, as well as nonadditive effects, and the crop-specific (in)breeding cycle (Jonas and De Koning 2013).

The reasons for considering spatial variation in crop breeding activities are obvious. Every trial (or environment) will have considerable sources of nongenetic variation such that even the position of a line in the field will impact its phenotypic response. Allowing for spatial variation through appropriate trial design and analysis will ensure that more accurate genetic effects are revealed (Gilmour *et al.* 1997). In GS, this is also true; the accuracy of genomic prediction in RR-BLUP is improved after adjusting for spatial variation using moving-means as a covariate in the model (Lado *et al.* 2013).

Similarly, consideration should be paid to the fact that crop lines are often assessed in a multi environment trial (MET), *i.e.*, in different geographic locations, seasons, or years, in order to determine performance stability across environments (*i.e.*, G × E). In GS, G × E is an important component of genetic variability (Crossa *et al.* 2010, 2011). A MET in a GS context is therefore an important extension as it allows the examination of marker by environment (M × E) interactions, and, in particular, the identification of markers whose effects are stable across environments (trials), as well as those that are environment-specific. As RR-BLUP involves fitting a linear mixed model, the incorporation of a MET extension is straightforward, and improvements in prediction when using two stage approaches to MET analysis have already been shown (Burgueno *et al.* 2012; Guo *et al.* 2013).

Capturing nonadditive effects in genomic selection is more complicated because the genomic relationship matrix described by the markers and used in RR-BLUP captures not only additive genetic relationships at QTL but also LD and cosegregation information (Habier *et al.* 2013). A first step toward modeling nonadditive effects in GS is to include pedigree information that captures a polygenic effect. Pedigree information included in a BayesB model in animal GS marginally improved the accuracy of selection, and reduced bias, which is important when marker effect estimates are used over multiple generations (Solberg *et al.* 2009). A small improvement in crops has also been shown (Crossa *et al.* 2010). Burgueno *et al.* (2012) explored the inclusion of pedigree information in RR-BLUP MET models, and found that it improved prediction accuracy for individual lines in some circumstances, but not others. However, compared to use of pedigree, inclusion of both additive and nonadditive marker-based, or realized genomic relationship matrices further improves prediction of breeding values (Munoz *et al.* (2014).

Most methods of crop GS use a two-stage analysis. First, data from individual replicated plants or plots are used to derive the line means. This allows software already developed for animal studies, which cannot handle replicates, to use the means for RR-BLUP in the second stage. However, a two-stage approach biases marker effects, and induces heterogeneous residual variances and residual correlations that are not completely eliminated by a weighted analysis (de los Campos *et al.* 2013). A single-stage approach that uses individual plant or plot data, includes replication, and accounts for spatial variation and randomization-based terms (*e.g.*, blocking factors), would be preferable because it would not have the difficulties associated with a two-stage approach. Incorporating data from individual plant or plot replicates would have additional advantages for GS. Markers may not capture all the genetic variation contributing to the phenotype, particularly if the number of markers used for prediction is low. Including replicates allows the total genetic effect due to lines to be partitioned into the genetic effect due to markers, and a residual genetic effect not captured by markers, which will include nonadditive genetic effects. This approach is possible without the need for pedigree information (which is not always available), would be more encompassing than a polygenic effect, and would not require the calculation of additional matrices that can induce dependency between variance components. In addition, separating out the nonadditive genetic effects from the model residual variance should increase the accuracy and predictive ability of the additive genetic effects compared to two-stage analyses.

In this paper, we propose such a single-stage approach to the analysis of multi-environment data by fitting a single linear mixed model that extends single trial RR-BLUP analysis. Marker and M × E interactions are incorporated as terms in the model, and form the basis for the analysis of METs. In this GS approach, the marker and M × E interaction terms are assumed to be random using RR-BLUP, and the approach extends RR-BLUP analysis by partitioning the term for genetic variation into marker and nonmarker (or residual genetic) variation using raw data from individual replicates, rather than line means. We first outline the proposed method that we refer to as *partitioned* RR-BLUP for MET, and then illustrate its use on a real data set with appropriate comparisons to nonpartitioned, or *standard*, models and single trial RR-BLUP analysis, both within a single-stage approach.

## Materials and Methods

### Description of motivating example

The new approach is illustrated with an example data set of the phenotypic trait ‘height’ in an association mapping population of cultivated barley. During two consecutive years (2010 and 2011; referred to also as trials), spring barley lines were grown in pots, in the field, within a polythene tunnel, with each pot containing one plant (from one line). In each trial, the pots were arranged in a spatial row-column design with five replicate blocks, where the replicate blocks correspond to biological replicates. In the 2010 trial, 648 lines were planted with pots arranged in 405 columns by eight rows, with each replicate block consisting of 81 columns by eight rows. In the 2011 trial, 856 lines were planted with pots arranged in 535 columns by eight rows, with each replicate block containing 107 columns (see Oakey *et al.* 2013 for further details). There were 639 lines common to both trial years. The lines were predominantly European elite cultivars of two-row spring barley. At full maturity, the height of each plant was measured in centimeters from the base of the plant to the top of the main stem. The software CycDesigN 4.0 (VSN International) was used to generate the design each trial year.

A set of 7864 high-confidence, gene-based single nucleotide polymorphism (SNP) markers, incorporated into a single Illumina iSelect assay (Illumina Inc.), was used to genotype DNA extracted from 477 lines (Comadran *et al.* 2012), including 459 lines grown across both years, one line from 2010 only, and 17 lines from 2011 only. Nonpolymorphic SNPs, and SNPs with more than 20% missing values, were removed. For each marker, individuals were coded as 0 (homozygous minor allele), or 2 (homozygous major allele). The population consists of lines that are derived via single seed descent and should be homozygous; a heterozygous marker within a particular individual suggests that there is an error with the calling of the marker, thus these heterozygous markers were coded as missing. Missing values were imputed using the R package impute (Hastie *et al.* 2014). Markers that were heterozygous and failed to converge to either 0 or 2 after several iterations were discarded, because this suggests the marker itself may not be appropriate for use. This resulted in a set of 4654 homozygous markers with a minimum minor allele frequency of >5% and map positions available for analysis. The final set of 3490 markers used in the analysis was a subset of the 4654 markers, and had no two markers identical in terms of their qualitative coding across the lines. Pedigree information on the lines was unavailable. The phenotypic and genotypic data are available in Supplemental Material, File S2.

### General form of the new model for whole genome prediction

A general form of the new model is now presented. This is a single linear mixed model that incorporates marker and M × E interactions as terms in the model with appropriate variance-covariance structures to allow for correlation between trials. In this new approach, the genetic variation is partitioned into marker and nonmarker (or residual) variation through the inclusion of all raw data in the model. In addition, spatial trends and design and randomized factors can be easily incorporated in the model. The new model is referred to as the *partitioned* RR-BLUP for MET. We use the term ‘trial’ to denote different environments, which for our example data set represents different years.

Consider a data set consisting of *v* lines and *s* trials. The new mixed model for whole genome prediction can be developed as follows

$$y=X\text{τ}+Z_{\text{g}}g+Z_{\text{u}}u+\varepsilon$$ (1)

where $y^{\left(n\times 1\right)}={\left(y_{1}^{T},\ldots ,y_{\text{s}}^{T}\;\right)}^{T}\;$is the vector of response across each of the *s* trials,$y_{t}^{\left(n_{t}\times 1\right)}$ is the vector of response for trial *t* and $n=\overset{s}{\underset{t=1}{\sum }}n_{\text{t}}$, where $n_{t}$ is the number of observations (pots) in trial *t*, **τ** is a vector of fixed terms, consisting of an overall mean performance for each trial, as well as trial specific global or extraneous spatial terms, for example, linear row or linear column effects, and ***X*** is the associated design matrix, $g^{\left(vs\;\times \;1\right)}$ is the vector of random line effects of the *v* lines in each of the *s* trials with design matrix $Z_{\text{g}}$, and has the general form,

$$g=\left(I_{\text{s}}⊗M\right)u_{\text{m}}+u_{\text{e}}$$ (2)

**M** is the (v×p) matrix of *v* lines by *p* SNP markers, $I_{s}$ is the (s×s) identity matrix, $u_{\text{m}}^{\left(ps\;\times \;1\right)}$is the vector of *p* random SNP marker effects in each of *s* trials and $u_{\text{e}}^{\left(vs\times 1\right)}$ is the vector of *v* random residual genetic effects in each of *s* trials, and represents line variation (and therefore genetic variation) that has not been accounted for by the markers; ⊗ is the kronecker product.

Let $u_{\text{m}}$ take a general form

$$u_{\text{m}}=\Lambda_{\text{m}}f_{\text{m}}+\delta_{\text{m}}$$ (3)

Where $\Lambda_{\text{m}}$ is a (ps×pk) matrix, $f_{\text{m}}^{\left(pk\;\times \;1\right)}$ is a vector with var($f_{\text{m}}$) = $G_{\text{m}}⊗I_{p}$, where $G_{\text{m}}$ is a (k×k) matrix for *k* factors, $\delta_{\text{m}}^{\left(ps\times 1\right)}$ is a vector with var$(\delta_{\text{m}}$) = $\Psi_{\text{m}}⊗I_{p}$ where $\Psi_{\text{m}}$ is the (s×s) marker genetic variance matrix across trials.

Let $u_{\text{e}}$ also take a general form

$$u_{\text{e}}=\Lambda_{\text{e}}f_{\text{e}}+\delta_{\text{e}}$$ (4)

Where $\Lambda_{\text{e}}$ is (vs×vl) matrix, $f_{\text{e}}^{\left(vl\;\times \;1\right)}$ is a vector with var($f_{\text{e}}$) = $G_{\text{e}}⊗I_{v}$ where $G_{\text{e}}$ is a (l×l) matrix for *l* factors, $\delta_{\text{e}}^{\left(vs\;\times \;1\right)}$ is a vector with var$(\delta_{\text{e}}$) = $\Psi_{\text{e}}⊗I_{v}$ where $\Psi_{\text{e}}$ is the (s×s) residual genetic variance matrix across trials.

Thus with *s* trials, the genetic variance matrices $\Psi_{\text{m}}$ and $\Psi_{\text{e}}$ are both (s×s) matrices, each with $\frac{s(s+1)}{2}$ parameters to be estimated.

Let $\Lambda_{\text{m}}=\Lambda_{\text{m1}}⊗I_{p}$ and $\Lambda_{\text{e}}=\Lambda_{\text{e1}}⊗I_{v}$, where $\Lambda_{\text{m1}}$ and $\Lambda_{\text{e1}}$ are (s×k) and (s×l) matrices of *k* and *l* factor loadings for each trial, respectively

then

$$\text{var}\left(u_{\text{m}}\right)=\left(\Lambda_{\text{m1}}G_{\text{m}}\Lambda_{\text{m1}}^{\text{T}}+\Psi_{\text{m}}\right)⊗I_{p}$$

and

$$\mathrm{var}\left(u_{\text{e}}\right)=\left(\Lambda_{\text{e1}}G_{\text{e}}\Lambda_{\text{e1}}^{\text{T}}+\Psi_{\text{e}}\right)⊗I_{v}$$

The vector $u^{\left(b\;\times \;1\right)}$ consists of sub vectors $u_{\text{i}}^{\left(b_{\text{i}}\;\times \;1\right)}$, where the subvector $u_{\text{i}}$ corresponds to the *i*th random term. The corresponding design matrix$\;Z_{\text{u}}^{\left(b\;\times \;1\right)}$ is partitioned conformably as $\left[Z_{u_{1}}\ldots Z_{u_{b}}\right]$. The subvectors are assumed mutually independent with variance ${\text{θ}}_{\text{i}}^{\text{2}}I_{b_{\text{i}}}$. The subvectors include random terms for describing spatial trends in individual trials, such as random row, random column, or spline terms. The residual vector **ε** has variance $R={⊕}_{t=1}^{s}R_{t}$, a block diagonal matrix of *s* blocks, $R_{t}={\text{θ}}_{\text{t}}^{\text{2}}I_{n_{\text{t}}}$.

In crops, the modeling of spatial trends in field trials is crucial (Gilmour *et al.* 1997), and the model above enables the addition of these trends where necessary. Furthermore, trial-specific design or randomization-based terms such as blocking factors can also be included in the model (Cullis *et al.* 2006).

Thus the line term ***g*** reflects the total genetic variation partitioned into additive variation as described by the markers and residual genetic or nonadditive variation, the fixed **τ**, random ***u*** and residual **ε** terms reflect the design and conduct of the trials, and as such provide the underlying structure for nongenetic variation.

#### Special cases of the general form of g:

The general form of ***g*** (Equation 2) shows the details for partitioning the total genetic variation. There are two special cases of ***g***. A *phenotypic* model can be fitted by letting $g=u_{\text{e}}$, so that the $u_{\text{m}}$ marker term is omitted, and ***g*** represents total genetic variation (assuming unrelated lines) as described by the phenotypic information. A *standard* RR-BLUP model for GS can be fitted by letting $g=\left(I_{s}⊗M\right)u_{\text{m}}$, so that $u_{\text{e}}$, the residual genetic term, is omitted; here, ***g*** represents additive genetic variation as described by the markers. The *standard* model reflects the most common current practice in GS, where only the markers are included in the model. These two additional models will be used as comparators to the new *partitioned* model.

#### Special cases of the general form of ${\text{u}}_{\text{m}}$:

From the general form of $u_{\text{m}}$ (Equation 3), special cases of *s*, the number of trials, and *k*, the number of factors, can be considered. By varying *s*, single and multiple environment trials are encompassed, and by varying *k* different structures for the variance-covariance matrix of $u_{\text{m}}$ can be considered (Table 1). Varying *k* enables an appropriate form of the var$\left(u_{\text{m}}\right)$ to be established to describe the correlation structure between trials, and may vary depending on the data set. A similar table of special cases of the form of $u_{\text{e}}$ (Equation 4) can also be constructed if l=k, e=m and v=p.

**Table 1:** Summary of the special cases of the general form of $u_{m}$*^a^*

Model	Description	*s*	*k*	$\Lambda_{m\text{1}}$	$G_{m}$	$\Psi_{m}$	STY or MET	Reference
Single trial	Diagonal (*s* = 1)	1	0			${\text{θ}}_{m}^{\text{2}}$	STY	
DIAG	Diagonal	*s*	0			${⊕}_{t\text{=1}}^{s}{\text{θ}}_{mt}^{\text{2}}$*^b^*	STY	
US	Unstructured	*s*	0			$\Psi_{m}$	MET	
CS	Compound symmetry	*s*	1	$1_{s}$	${\text{θ}}_{m}^{2}$	${\text{θ}}_{m\text{e}}^{\text{2}}I_{\text{s}}$	MET	Patterson *et al.* (1977)
CS+DIAG	CS+DIAG	*s*	1	$1_{s}$	${\text{θ}}_{m}^{\text{2}}$	${⊕}_{t=1}^{s}{\text{θ}}_{mt}^{\text{2}}$	MET	Cullis *et al.* (1998)
FAM*k**^c^*^,^*^d^*	Factor analytic (main effect)	*s*	k+1	$\left[{\text{θ}}_{m}1_{s}\;\Lambda_{f}\right]$	$\left[\begin{array}{cc} {\text{θ}}_{m}^{\text{2}} & 0\\ 0 & I_{k} \end{array}\right]$	${⊕}_{t=1}^{s}{\text{θ}}_{mt}^{\text{2}}$	MET	Smith *et al.* (2001)

STY, single trial year (note the DIAG model is equivalent to analyzing each trial year separately); MET, multi-environment trial.

a A similar table could be constructed for $u_{e}$ with l=k, e=m and v=p.

b ⊕ represents a kronecker sum, so that ${⊕}_{t=1}^{s}{\text{θ}}_{mt}^{\text{2}}$ results in a diagonal matrix with elements ${\text{θ}}_{mt}^{\text{2}}$ for the specific variance of trial *t*.

c $\Lambda_{f}^{\left(s\;\times \;k\right)}$ is a matrix of *k* factor loadings at each of the *s* trials.

d For FAM*k* let $f_{m}^{T}=(f_{0}^{\text{T}},f^{\text{T}})$ where $u_{0}={\text{θ}}_{m}f_{0}$, var$(u_{0})={\text{θ}}_{m}^{\text{2}}I_{\text{p}}$ and $\mathrm{var}\left(f\right)=I_{k}⊗I_{p}$.

For a single trial $u_{\text{m}}^{\left(p\;\times \;1\right)}=\delta_{\text{m}}^{\left(p\;\times \;1\right)}$ is a main marker term, with $\text{var}(u_{\text{m}})={\text{θ}}_{\text{m}}^{2}I_{p}$. Models for multiple environment trials are now discussed.

The simplest model for more than one trial is the diagonal (DIAG) model, where $u_{\text{m}}^{\left(sp\;\times \;1\right)}=\delta_{\text{m}}^{\left(sp\;\times \;1\right)}$ is a main marker term in each of the trials. The $\text{var}\left(u_{\text{m}}\right)=\Psi_{\text{m}}⊗I_{p}$, where $\Psi_{\text{m}}$ has off-diagonals for all trials assumed zero. The DIAG model therefore assumes a separate marker variance for each trial, and no marker covariance between trials, and is equivalent to fitting each trial separately.

In the compound symmetry (CS) model, $f_{\text{m}}^{\left(p\;\times \;1\right)}$ is a main term for markers, and $\delta_{\text{m}}^{\left(ps\;\times \;1\right)}$ is an interaction term for the markers and trials. All trials have the same marker variance, and all pairs of trials have the same marker covariance, so that the $\text{var}\left(u_{\text{m}}\right)=\left({\text{θ}}_{\text{m}}^{2}J_{s}+\Psi_{\text{m}}\right)⊗I_{p}$ where $J_{s}$ is (s×s) matrix of ones and $\Psi_{\text{m}}={\text{θ}}_{\text{me}}^{2}I_{s}$.

For the Cullis *et al.* (1998) (CS+DIAG) model, $f_{\text{m}}^{\left(p\;\times \;1\right)}$ is a main marker term, and $\delta_{\text{m}}^{\left(ps\;\times \;1\right)}$ is a term for the interaction of the markers and trials. This model assumes the same marker covariance for pairs of trials, and a separate marker variance for each trial. Thus, the form of $\text{var}(u_{\text{m}})$ is the same in the CS and (CS+DIAG) models; however, in the latter, $\Psi_{\text{m}}={⊕}_{t=1}^{s}{\text{θ}}_{\text{m}t}^{2}$. In this model, the covariance between pairs of trials is assumed to be not greater than the variance of the individual trials.

An unstructured (US) model allows different marker variances and covariances between trials, so that $u_{\text{m}}^{\left(sp\;\times \;1\right)}=\delta_{\text{m}}^{\left(sp\;\times \;1\right)}$ is an interaction term of the markers and trials, and no main marker effect is fitted. The$\text{ var}\left(u_{\text{m}}\right)=\Psi_{\text{m}}⊗I_{p}$, where $\Psi_{\text{m}}$ has diagonal elements that are the marker variances for the individual trials, and off-diagonal element that are the marker covariance between trials. As the number of trials increases, the US model becomes over parameterized, making it difficult to fit.

Multiplicative models have been shown to work well in practice in MET analysis (Smith *et al.* 2005), and are viable alternatives to the unstructured model. In fact, Kelly *et al.* (2007) found that the factor analytic model with *k* factors (FA*k*) of Smith *et al.* (2001) was preferred over an unstructured model because it improved the predictive accuracy of the line empirical BLUPs.

In a GS situation, a factor analytic model with a main marker term and *k* factors (FAM*k*) may be more appropriate than a FA*k* model that excludes this term. This is because a main marker term represents QTL that are common and stable across trials (in the absence of an interaction between trials and markers) and the marker × trial term will give information on QTL that are trial or environment specific.

Smith *et al.* (2001) showed that a FAM*k* model is equivalent to a factor analytic model with (*k*+1) factors, where the first set of loadings are constrained to be equal. For a FAM*k* model, we let $f_{\text{m}}^{\text{T}}=(f_{0}^{\text{T}},f^{\text{T}})$, where $u_{0}={\text{θ}}_{\text{m}}f_{0}$, with var$(u_{0})={\text{θ}}_{\text{m}}^{2}I_{p}$, $f^{\left(sk\;\times \;1\right)}$ is a vector of line scores with $\mathrm{var}\left(f\right)=I_{k}⊗I_{p}$, then $\text{var}\left(u_{\text{m}}\right)=\left({\text{θ}}_{\text{m}}^{2}J_{s}+\Lambda_{f}\Lambda_{f}^{\text{T}}+\Psi_{\text{m}}\right)⊗I_{\text{p}}$, where $\Lambda_{f}$ is a (s×k) matrix of loadings, and $\Psi_{\text{m}}$ is a diagonal matrix with the diagonal elements referred to as specific variances. The approach of including a main marker term is in contrast to a phenotypic model for estimating genotypic values, where, in crop field trials, the main line term is usually excluded. Notice when *k = 0*, the FAM*0* model is equivalent to the CS+DIAG model. The special cases of the general form of $u_{\text{m}}$ shown in Table 1, can be fitted in each of the three models, *phenotypic*, *standard*, and *partitioned*, which reflect different forms of ***g*** (Equation 2).

#### Computational efficiency:

If the number of markers exceeds the number of individuals, we can fit

$$u_{\text{g}}=\left(I_{\text{s}}⊗M\right)u_{\text{m}}$$

The analysis will now be dependent on the number of lines rather than on the number of markers, and therefore will reduce the dimensionality of the model, making it more computationally efficient (Stranden and Garrick 2009).

The estimation of variance parameters is by residual maximum likelihood (REML). Given estimates of the variance components, empirical best linear unbiased predictors (E-BLUPs) were obtained for random terms from the mixed model equations as

$${\tilde{u}}_{\text{m}}=\left(I_{\text{s}}⊗M_{\text{T}}{\left({MM}^{T}\right)}^{-1}\right){\tilde{u}}_{\text{g}}$$ (5)

where ${\tilde{u}}_{\text{g}}$ is the vector of genomic breeding values in each trial.

Here, we use ${MM}^{T}$as in Piepho *et al.* (2012), where ${MM}^{T}$ represents a realized genomic relationship matrix. Meuwissen *et al.* (2001) used ${MM}^{T}/p$, where *p* is the number of marker (locations), and Habier *et al.* (2007) used ${MM}^{T}/2\underset{q}{\sum }p_{q}(1-p_{q})$, where $p_{q}$ is the allele frequency at marker locus *q*. Omission of the scalar term will not affect the conclusions of the analysis.

If ${\left({MM}^{T}\right)}^{-1}$ is of full rank, then

$$\text{var}\left(\begin{array}{c} u_{\text{g}}\\ u_{\text{m}} \end{array}\right)=G_{f}⊗\left[\begin{array}{cc} {MM}^{T} & M\\ M^{T} & I_{\text{p}} \end{array}\right]$$

for $G_{f}=\Lambda_{\text{m1}}G_{\text{m}}\Lambda_{\text{m1}}^{\text{T}}+\Psi_{\text{m}}$

Thus, if

$$u_{\text{g}}=\left(I_{s}⊗M\right)u_{\text{m}}=\left(I_{s}⊗M\right)\Lambda_{\text{m}}f_{\text{m}}+\left(I_{s}⊗M\right)\delta_{m}=u_{f}+u_{\text{δ}}$$

Then, the E-BLUPs obtained from the mixed model equations are

$$\Lambda_{m}{\overset{˜}{f}}_{\text{m}}=\left(I_{s}⊗M^{T}{\left(MM^{T}\right)}^{-1}\right){\overset{˜}{u}}_{f}$$ (6)

and

$${\tilde{\delta }}_{\text{m}}=\left(I_{s}⊗M^{T}{\left(MM^{T}\right)}^{-1}\right){\tilde{u}}_{\delta }$$ (7)

If ${{(MM}^{T})}^{-1}$ is of full rank, then

$$\text{var}\left(\begin{array}{c} u_{f}\\ \Lambda_{\text{m}}f_{\text{m}} \end{array}\right)=\Lambda_{\text{m1}}G_{\text{m}}\Lambda_{\text{m1}}^{\text{T}}⊗\left[\begin{array}{cc} {MM}^{T} & M\\ M^{T} & I_{p} \end{array}\right]\;\text{and}\;\text{var}\left(\begin{array}{c} u_{\text{δ}}\\ \delta_{\text{m}} \end{array}\right)=\Psi_{\text{m}}⊗\left[\begin{array}{cc} {MM}^{T} & M\\ M^{T} & I_{p} \end{array}\right]$$

For our data set, the number of markers exceeds the number of individuals, therefore the *standard* and *partitioned* models were fitted using the computationally efficient approach. All models were fitted in ASReml v3.0-1 (Butler *et al.* 2009) for R v3.2.0 (R Core Team 2015). Instructions for completing all the analysis shown in the paper can be found in File S1 along with supporting data (File S2) and R scripts (File S3, File S4, File S5, File S6, File S7, File S8, File S9, File S10, File S11, File S12, File S13, File S14, File S15, File S16, File S17, File S18, and File S19).

### Heritability

The calculation of the generalized heritability in complex linear mixed models is not straightforward (Cullis *et al.* 2006). Here, the generalized heritability for each trial is calculated from the phenotypic model (where $g=u_{\text{e}}$) as $1-\frac{a}{2{\text{θ}}_{\text{g}t}^{2}}$ where *a* is the average pairwise prediction error variance of line effects, and ${\text{θ}}_{\text{g}t}^{\text{2}}$ is the genetic variance of trial *t* (Cullis *et al.* 2006). The R code for calculating the heritability is in File S19.

### Cross-validation

Initially, the *phenotypic*, *standard*, and *partitioned* models were fitted using the *full* data set to enable the most appropriate MET form of $u_{\text{m}}$ (Table 1), the term representing marker or additive variation, and $u_{\text{e}}$, the term representing nonadditive or residual genetic variance, to be established for use in the cross-validation. A single trial analysis was also investigated in the cross-validation represented by the DIAG form. We generally denote the *phenotypic*, *standard*, and *partitioned* models with specific forms of $u_{\text{m}}$ as E_FORM_, S_FORM_, and P_FORM_, respectively [where FORM is either DIAG, US, CS, CS+DIAG, or FAM*k* (Table 1)].

To establish which MET form of $u_{\text{m}}$ is superior, and to undertake initial comparisons between the three models, the Akaike information criteria (AIC) (Akaike 1974), or log-likelihood ratio test (if the models were nested), were used. For example, the *phenotypic* and *standard* model are nested within the *partitioned* model when the form of $u_{\text{m}}$ is the same, and can be compared using the log-likelihood ratio test. However, models with different forms for $u_{\text{m}}$ need to be compared using the AIC. The AIC was calculated as twice the number of random parameters minus twice the log-likelihood (the number of fixed parameters was ignored in this calculation as this was constant over the models). For the AIC, lower values indicate superior models. Once the form of $u_{\text{m}}$ for the MET analyses has been established, the cross-validations could proceed.

The cross-validation involved randomly dividing the lines in the data set (as evenly as possible) into 10 groups, which were used across the single and MET analyses so that consistency and comparability was maintained as much as possible (Table 2).

**Table 2:** Number of lines with marker information in the groups used in the cross-validation

Groups for Cross-Validation	Number of Lines*^a^* in Each Group			Total Number of Lines in Each Group (Total Number of Lines Across Groups)		
	Common*^b^*	2010 Only	2011 Only	2010	2011	MET*^c^*
1–6	46	0	2	46 (276)	48 (288)	48 (288)
7–9	46	0	1	46 (138)	47 (141)	47 (141)
10	45	1	2	46 (46)	47 (47)	48 (48)
Total	459	1	17	460	476	477

a These are the number of lines with marker information.

b The common lines groups are kept the same across all analyses.

c The multi-environment trial (MET) analyses contain information from both trial years.

Three different cross-validations were examined, and were defined by the number of groups in the validation and training sets. These were CV10, CV20, and CV40, where approximately 10, 20, and 40% of lines, respectively, were included in the validation set (Table 3), with the remaining lines used as the training set. Iteration across all combinations was investigated. For example, for the CV20 cross-validation, two groups (the validation set) were omitted in any one iteration. To cover the possible combinations of two of the 10 groups, a total of 45 iterations were necessary. The R scripts with details of the random division of the data and group combinations for CV10, CV20, and CV40 are in File S3, File S4, and File S5, respectively.

**Table 3:** Summary of validation and training groups in three cross-validations

Cross-Validation	Number of Groups in VALIDATION Set	Number*^a^* (%) of Line Numbers in the VALIDATION Set			Number of Groups in TRAINING Set	Total Number*^b^* of Iterations
		2010	2011	MET (both years)		
CV10	1	46 (10)	47 (9.7) – 48 (10.1)	47 (9.9) – 48 (10.1)	9	10
CV20	2	92 (20)	94 (19.7) – 96 (20.2)	94 (19.7) – 96 (20.1)	8	45
CV40	4	184 (40)	188 (39.5) – 192 (40.3)	188 (39.4) – 192 (40.3)	6	210

a The number will be a range for 2011 and the MET as the number of lines in each group (Table 2) is variable.

b This is the number of iterations so all combinations of groups in the validation set can be investigated.

Using the training set, marker effects were obtained under each model (*standard* and *partitioned*), each cross-validation (CV10, CV20, and CV40), and each scenario (single trial 2010, single trial 2011, and MET). The marker effects (Equation 6 and Equation 7) were used to predict the GEBVs of the lines in the validation set. The DIAG form of $u_{\text{m}}$ and $u_{\text{e}}$ was used to generate marker effects equivalent to analyzing each trial separately, with each trial having one set of marker effects. For the MET analyses of both trials, different forms of $u_{\text{m}}$ and $u_{\text{e}}$ (Table 1) were initially investigated as described previously, with the most appropriate forms used in the cross-validation. For the CS, CS+DIAG, and FAM*1* forms of $u_{\text{m}}$ and $u_{\text{e}}$, there were three sets of marker effects. These were: the main marker effects, representing markers that are stable across both trials, and marker effects from each trial, which represent the additional marker by trial interaction. A total marker effect for each trial was obtained by adding the main marker effect to the marker by trial interaction effect. For completeness, the US form, which produces only total marker effects for each trial, was also initially investigated, although, as we are interested in MET models with main marker effects, the cross-validation focused on the most appropriate of the CS, CS+DIAG, and FAM*1* forms.

In simulations, the true breeding value is known, and the GEBV calculated in the lines of the validation set can be compared to the true breeding values to determine the predictive ability and the accuracy and precision of the GEBV. As real data were used here, the true breeding value was unknown. In the absence of pedigree information, the genotypic values (GV) of the lines in the validation set were used as the comparator. These were calculated from a *phenotypic* model, based on all the lines with marker information, where lines were assumed to be unrelated. The GVs from this *phenotypic* model reflect total genetic effects, and were calculated using single trial and MET analyses. In crops, breeders are most often interested in the commercial potential of lines, so the adoption of the genotypic values (which are the total genetic values of each line) as the comparator to GEBV of validation lines reflects this crop breeding strategy. Linear regression models were fitted in which the response was the observed GVs of lines in the validation set, and the explanatory variable was the GEBVs of those same lines calculated using the marker effects. The cross-validation investigated the performances of the *partitioned* and *standard* RR-BLUP models in terms of their predictive ability measured by the R-squared value, and the accuracy and precision of the GEBV measured by the mean square error (MSE) from the linear regression models. The *standard* and *phenotypic* models were compared using the same training sets.

Comparisons were investigated between the GEBVs and GVs, where the same model (single trial or MET) and marker terms were used to derive both the GEBV and GV. A further comparison was made between the GEBVs of the main effect from the MET model to GVs from a single trial analysis of each trial year. The R script for performing each cross-validation (CV10, CV20, and CV40) under each scenario (single trial 2010, single trial 2011, and MET) can be found in File S6, File S7, File S8, File S9, File S10, and File S11.

### Implementing GS with a lower density of markers

In addition to using all the markers to predict the GEBV of lines in the validation set, we explored a low-density GS approach, where a subset of *random*, rather than significant, markers and their effects were used to predict the GEBV of lines. This was explored for CV10 only (Table 2).

For each of the 10 training sets of lines, subsets of markers of increasing size, *x*, from *x* = 10 to *x* = 3490 (all markers) were chosen at random. For each size *x*, and each training set of lines, the markers were chosen at random, and resampled to provide 200 different random combinations of size *x*. The performance at each subset of markers was measured as the average of the regression coefficients, and mean square error over the 200 random combinations of size *x*. For each *x*, each scenario and model was compared using the same random marker subsets and the same training set of lines. R scripts can be found in File S12, File S13, File S14, File S15, File S16, and File S17.

### Data availability

All data necessary for confirming the conclusions presented in this manuscript are provided in Supplemental Material.

## Results

### Height phenotypic data in multi-environment trials

**Figure 1:**
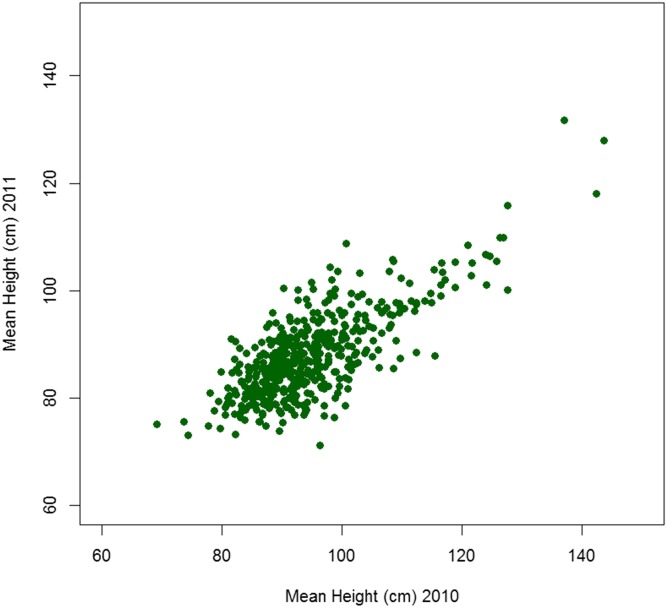
Correlation of mean heights of lines in the 2010 and 2011 trials. The datapoints represent only the lines with marker data that were grown in both years.

An association mapping population of two-row spring barley lines was grown in a randomized spatial row-column design in two consecutive trials in years 2010 and 2011 (*i.e.*, two environments), with five replicate plants of each line. At full maturity, the height of each plant was measured. The raw mean heights for lines grown in both years, and for which we have marker information, are shown in Figure 1. The plant height was slightly elevated in 2010 as opposed to 2011; the mean height in 2010 was 94.8 cm, and in 2011 was 87.9 cm. The correlation between the means of the line heights across the years was high at 0.76. These data were subsequently used, as described below, to develop our new GS model, and to predict, based only on the complement of lines with molecular markers present in each training set, the GEBV for height of lines that were in each validation set.

### Comparison of models for whole genome prediction

Initially, the *partitioned*, *standard*, and *phenotypic* models were fitted (Table 4) using the full data set, enabling the form of $u_{\text{m}}$ (Table 1), the term representing marker variation, and $u_{\text{e}}$, the term representing nonadditive or residual genetic variance, to be established for use in the cross-validation. All models included a random term for replicate block for both trials; an additional random term was also included in the analysis to account for spatial variation present between columns in 2011.

**Table 4:** Summary of the models fitted to the full data set

Model*^a^*		Form*^b^* of ***u***_m_	Form of ***u***_e_	STY or MET	Log-Likelihood	AIC
Phenotypic*^c^*	E_DIAG_		DIAG*^d^*	STY	−11,074.6	22,163.1
	E_CS_		CS*^e^*	MET	−10,877.0	21,768.1
	E_CS+DIAG_		CS+DIAG*^f^*	MET	−10,872.5	21,760.9
	E_FAM_*_1_*, E_US_		FAM*1**^g^*, US*^h^*	MET	−10,872.4	21,760.8
Standard*^i^*	S_DIAG_	DIAG		STY	−10,924.9	21,863.8
	S_CS_	CS		MET	−10,794.0	21,602.1
	S_CS+DIAG_	CS+DIAG		MET	−10,790.6	21,597.2
	S_FAM_*_1_*, S_US_	FAM*1*, US		MET	−10,787.8	21,591.6
Partitioned*^j^*	P_DIAG_	DIAG	DIAG	STY	−10,876.4	21,770.8
	P_CS_	CS	CS	MET	−10,747.2	21,512.5
	P_CS+DIAG_	CS+DIAG	CS+DIAG	MET	−10,744.6	21,511.2
	P_FAM_*_1_*, P_US_	FAM*1*, US	FAM*1*, US	MET	−10,744.2	21,510.4

STY, single trial year; MET, multi-environment trial; AIC, Akaike information criteria.

a All models derive from Equation 1 but are special cases of ***g***(Equation 2).

b Details of forms of ***u***_m_ are given in Table 1.

c Phenotypic model has ***g*** = ***u***_e_.

d DIAG implies the covariance between the two trials is assumed to be zero, and is equivalent to fitting the two trials separately.

e CS is the compound symmetry model.

f CS+DIAG is the model described by Cullis *et al.* 1998.

g FAM*1* is the factor analytic model (Smith *et al.* 2001), with main effect with *k* the number of factors equal to 1.

h US is the unstructured model (US), for two trials this model is equivalent to the FAM*1* model.

i Standard RR-BLUP model has $g=\left(I_{s}⊗M\right)$***u***_m_.

j Partitioned RR-BLUP model has $g=\left(I_{s}⊗M\right)u_{m}+u_{e}$.

Log-likelihood ratio tests comparing *standard* and *partitioned* models with the same form of $u_{\text{m}}$ (*e.g.*, S_DIAG_ *vs.* P_DIAG_, S_CS+DIAG_ *vs.* P_CS+DIAG_, etc.) were significant (*P* < 0.001), suggesting the *partitioned* models are superior to the *standard* models (Table 4). In addition, all the MET models had lower AIC than the single trial year models, suggesting that the MET models were superior (Table 4). For two trials, the factor analytic models (E_FAM_*_1_*, S_FAM_*_1_*, and P_FAM_*_1_*), and the unstructured models (E_US_, S_US_, and P_US_), respectively, are identical, and therefore the US results are shown with the FAM*1* model in Table 4. Given that the factor analytic model has been shown previously to improve the predictive accuracy of the line empirical BLUPs, that factor analytic models are easier to fit than an unstructured model (Kelly *et al.* 2007), and given that we are primarily interested in models that fit a main marker term, the US model is not discussed further.

For the *phenotypic*, *standard*, and *partitioned* MET models, the compound symmetry form (E_CS_, S_CS_ and P_CS_) had the highest AIC, suggesting that this model was not a good choice in comparison to the other forms. The CS+DIAG forms (E_CS+DIAG_ and P_CS+DIAG_) had similar AICs to the factor analytic models (E_FAM_*_1_* and P_FAM_*_1_*). However, the factor analytic model of the *standard* model (S_FAM_*_1_*) showed a lower AIC than the CS+DIAG form (S_CS+DIAG_). Examining the REML estimates of the variance components (Table 5), it is clear that the CS+DIAG model does not necessarily fit as well as the FAM*1* model, as the covariance between years has been constrained to be equal to the 2011 trial variance. However, as the variance component estimates of the CS+DIAG form (E_CS+DIAG_, S_CS+DIAG_, and P_CS+DIAG_) and the FAM*1* form (E_FAM_*_1_*, S_FAM_*_1_*, and P_FAM_*_1_*) were similar for this data despite these constraints, both these forms were investigated further in the cross-validation. It is worth noting that Table 4 does not show the complete set of possible *partitioned* models. For the *partitioned* model, we have explored only models with the same form for the $u_{\text{m}}$ and $u_{\text{e}}$ terms. It is, however, possible to include different forms for each of $u_{\text{m}}$ and $u_{\text{e}}$ terms, for example, the former could take a compound symmetry form, and the latter a factor analytic form. When fitting the FAM*1* model, the full parameterization required five parameters (one for the main effect, two loadings, and two specific variances), two more than the three required. Thus, when fitting the FAM*1* model, the two specific variances were constrained to be zero. Smith *et al.* (2001) discuss the parameter constraints necessary for FA*k* models with *k* > 1. Thus based on the results of the log-likelihood ratio tests, AIC, and estimates of the model variance components, for the MET analysis of trials in the cross-validation, we explore only the CS+DIAG and FAM*1* forms for the marker and residual genetic effects. From the variance components of the diagonal form of the phenotypic model, the heritability of the trials was calculated as 0.90 in 2010 and 0.75 in 2011, so both trials have high heritability. The difference in heritability between years reflects a greater contribution of environment to the variation in 2011, and consequently a lower proportion of variation that is genetic in that year. A comparison between the variance of the $u_{\text{e}}$ term from *phenotypic* and the *partitioned* DIAG models (E_DIAG_ and P_DIAG_, respectively) enabled an estimate of the proportion of genetic variation accounted for by the markers, which was 0.75 for trial 2010, and 0.73 for trial 2011. The R code for determining the proportion of genetic variation is in File S19.

**Table 5:** REML estimates of variance components of the models fitted (Table 4)

		Form*^b^* of ***u***_m_	Form of ***u***_e_	STY or MET	var(u_m_)			var(u_e_)			Residual $R_{t}$	
Model*^a^*					Year 2010	Year 2011	Covar	Year 2010	Year 2011	Covar	Year 2010	Year 2011
Phenotypic	E_DIAG_		DIAG	STY				111.65	86.35	0	39.23	95.41
	E_CS_		CS	MET				103.22	103.22	92.77	39.37	93.73
	E_CS+DIAG_		CS+DIAG	MET				109.05	89.45	89.45	39.23	94.95
	E_FAM_*_1_*, E_US_		FAM*1*, US	MET				109.99	88.18	89.59	39.23	95.29
Standard	S_DIAG_	DIAG		STY	0.0703	0.0365	0				40.72	101.26
	S_CS_	CS		MET	0.0659	0.0659	0.0626				41.23	97.99
	S_CS+DIAG_	CS+DIAG		MET	0.0670	0.0595	0.0595				41.01	98.09
	S_FAM_*_1_*, S_US_	FAM*1*, US*^c^*		MET	0.0718	0.0519	0.0584				40.76	99.83
Partitioned	P_DIAG_	DIAG	DIAG	STY	0.0265	0.0194	0	27.67	23.43	0	39.24	95.73
	P_CS_	CS	CS	MET	0.0240	0.0240	0.0227	27.04	27.04	19.97	39.35	94.11
	P_CS+DIAG_	CS+DIAG	CS+DIAG	MET	0.0246	0.0218	0.0218	28.84	22.03	19.54	39.23	95.51
	P_FAM_*_1_*, P_US_	FAM*1*, US	FAM*1*, US	MET	0.0260	0.0205	0.0221	27.86	23.46	19.35	39.23	95.48

STY, single trial year; MET, multi-environment trial, Covar, covariance between trial year 2010 and trial year 2011.

a All models derive from Equation 1 but are special cases of g (Equation 2).

b Details of forms of ***u***_m_ are given in Table 1 and Table 4.

c For two trials the US model is equivalent to the FAM*1* model.

The variance components of the residual terms in each year were higher in the *standard* models compared to the equivalent forms of the *phenotypic* and *partitioned* models, particularly in 2011. The total genetic variation is therefore not accounted for in this model, perhaps as expected, given that the marker effect should reflect just additive genetic variation; the proportion of the genetic variation not being accounted for by the markers thus has contributed to the enlarged residual term. Given these limitations, the *standard* models were not taken forward for cross-validation.

### Cross-validation of selected models

The lines were randomly divided into 10 equivalent groups to facilitate comparative cross-validations, where the majority of lines were used as the training set, while 10%, 20%, or 40% of lines constituted the CV10, CV20, and CV40 validation sets, respectively (see *Materials and Methods* and Table 3).

The cross-validation focuses on comparing the *partitioned* and the *standard* RR-BLUP model in each of three scenarios, which are each trial year separately (P_DIAG_ and S_DIAG_, respectively), and in a joint MET analysis of both years. From the initial fitting of the MET models just discussed, we examine only the models where the form of $u_{\text{m}}$ and $u_{\text{e}}$ takes either CS+DIAG (P_CS+DIAG_ and S_CS+DIAG_, respectively), or FAM*1* (P_FAM_*_1_* and S_FAM_*_1_*, respectively). When fitting the *standard* and *partitioned* models of the factor analytic form (S_FAM_*_1_*, P_FAM_*_1_*) in ASReml, specific variances were set to zero as in the full data set, and the variance components of S_FAM_*_1_* were used as starting values for the variance components of P_FAM_*_1_*.

Various comparisons between the predicted (GEBVs) and observed (GVs) results were made, and the performance of the *partitioned* and *standard* RR-BLUP models in terms of predictive ability of the markers was measured by the R-squared value (Table 6), while the accuracy of line estimates (GEBVs) was measured by the mean square error (Table 7). Both of these were averages across the iterations (Table 3).

**Table 6:** Average *R*-squared*^a^* (SD) of partitioned verses standard RR-BLUP model for different cross-validation (Table 3), models (Table 4) and effects used to generate the GV and GEBV

						*R*-Squared (SD)					
						CV40		CV20		CV10	
Comparison	Phenotypic Model	Line Effects to Generate GV*^b^*	Standard Model	Partitioned Model	Marker*^c^* Effects to Generate GEBV	Standard Model	Partitioned Model	Standard Model	Partitioned Model	Standard Model	Partitioned Model
1	E_DIAG_	2010	S_DIAG_	P_DIAG_	**2010**	0.366(0.047)	0.406(0.048)	0.390(0.055)	0.438(0.064)	0.404(0.085)	0.461(0.093)
2	E_DIAG_	2010	S_DIAG_	P_DIAG_	**2011**	0.333(0.055)	0.359(0.062)	0.362(0.091)	0.383(0.095)	0.387(0.139)	0.407(0.137)
3	E_DIAG_	2011	S_DIAG_	P_DIAG_	**2011**	0.280(0.043)	0.298(0.046)	0.304(0.081)	0.318(0.081)	0.323(0.144)	0.334(0.138)
4	E_DIAG_	2011	S_DIAG_	P_DIAG_	**2010**	0.252(0.050)	0.288(0.043)	0.267(0.070)	0.307(0.060)	0.277(0.122)	0.319(0.097)
5	E_CS+DIAG_	Total 2010	S_CS+DIAG_	P_CS+DIAG_	***Total 2010***	0.368(0.044)	0.410(0.046)	0.392(0.049)	0.439(0.061)	0.406(0.078)	0.462(0.088)
6	E_CS+DIAG_	Total 2011	S_CS+DIAG_	P_CS+DIAG_	***Total 2011***	0.360(0.041)	0.401(0.043)	0.382(0.053)	0.428(0.062)	0.395(0.089)	0.448(0.098)
7	E_FAM*1*_	Total 2010	S_FAM*1*_	P_FAM*1*_	***Total 2010***	0.320(0.044)	0.376(0.051)	0.335(0.059)	0.403(0.071)	0.344(0.093)	0.423(0.097)
8	E_FAM*1*_	Total 2011	S_FAM*1*_	P_FAM*1*_	***Total 2011***	0.365(0.043)	0.402(0.048)	0.386(0.052)	0.430(0.056)	0.396(0.085)	0.448(0.083)
9	E_DIAG_	2010	S_CS+DIAG_	P_CS+DIAG_	***Main***	0.350(0.048)	0.392(0.056)	0.371(0.068)	0.419(0.082)	0.387(0.101)	0.442(0.115)
10	E_DIAG_	2011	S_CS+DIAG_	P_CS+DIAG_	***Main***	0.271(0.040)	0.302(0.040)	0.289(0.066)	0.322(0.068)	0.300(0.117)	0.336(0.115)
11	E_DIAG_	2010	S_FAM*1*_	P_FAM*1*_	***Main***	0.323(0.059)	0.377(0.063)	0.336(0.087)	0.404(0.092)	0.349(0.122)	0.430(0.125)
12	E_DIAG_	2011	S_FAM*1*_	P_FAM*1*_	***Main***	0.268(0.039)	0.299(0.040)	0.287(0.070)	0.323(0.070)	0.302(0.128)	0.338(0.118)

GEBV, genomic estimated breeding value; GV, genotypic value SD=standard deviation.

a The *R*-squared value is from a linear model for the validation set in which the GEBV is the covariate and the GV the response, the *R*-squared value shown is the average of the *R*-squared value over the different iterations (Table 2). Large *R*-squared values indicate better predictive ability.

b GV are calculated using a phenotypic model with all of the lines.

c Marker effects from DIAG form are in bold with year of trial shown, and are equivalent to results from a single trial year analysis, marker effects for the MET analyses are in bold and italic, three marker effects are possible: main, interaction 2010, interaction 2011; with the sum of the (main + interaction) marker effects being equivalent of a total marker effect for a particular year.

**Table 7:** Average mean square error*^a^* (SD) of partitioned verses standard RR-BLUP model for cross-validation (Table 3), models (Table 4) and effects used to generate the GV and GEBV

Comparison	Phenotypic Model	Line Effects to Generate GV*^b^*	Standard Model	Partitioned Model	Marker Effects to Generate GEBV*^c^*	Mean Square Error (SD)					
						CV40		CV20		CV10	
						Standard Model	Partitioned Model	Standard Model	Partitioned Model	Standard Model	Partitioned Model
1	E_DIAG_	2010	S_DIAG_	P_DIAG_	**2010**	8.13(0.74)	7.86(0.72)	7.96(1.09)	7.64(1.09)	7.85(1.59)	7.47(1.57)
2	E_DIAG_	2010	S_DIAG_	P_DIAG_	**2011**	8.27(0.55)	8.10(0.54)	8.04(0.77)	7.91(0.78)	7.82(1.05)	7.70(1.08)
3	E_DIAG_	2011	S_DIAG_	P_DIAG_	**2011**	6.85(0.40)	6.77(0.39)	6.72(0.60)	6.65(0.59)	6.57(0.90)	6.52(0.89)
4	E_DIAG_	2011	S_DIAG_	P_DIAG_	**2010**	7.06(0.51)	6.89(0.49)	6.98(0.73)	6.80(0.71)	6.92(1.09)	6.72(1.01)
5	E_CS+DIAG_	Total 2010	S_CS+DIAG_	P_CS+DIAG_	***Total 2010***	8.01(0.75)	7.74(0.70)	7.85(1.09)	7.53(1.04)	7.73(1.55)	7.35(1.49)
6	E_CS+DIAG_	Total 2011	S_CS+DIAG_	P_CS+DIAG_	***Total 2011***	7.02(0.57)	6.79(0.55)	6.88(0.77)	6.61(0.75)	6.75(1.02)	6.44(1.01)
7	E_FAM*1*_	Total 2010	S_FAM*1*_	P_FAM*1*_	***Total 2010***	9.98(0.87)	9.55(0.83)	9.87(1.39)	9.34(1.34)	9.80(2.07)	9.19(1.96)
8	E_FAM*1*_	Total 2011	S_FAM*1*_	P_FAM*1*_	***Total 2011***	7.02(0.62)	6.81(0.61)	6.88(0.87)	6.63(0.83)	6.79(1.22)	6.49(1.13)
9	E_DIAG_	2010	S_CS+DIAG_	P_CS+DIAG_	***Main***	8.06(0.63)	7.79(0.61)	7.89(0.87)	7.58(0.88)	7.75(1.17)	7.38(1.18)
10	E_DIAG_	2011	S_CS+DIAG_	P_CS+DIAG_	***Main***	6.87(0.42)	6.73(0.41)	6.78(0.59)	6.62(0.60)	6.69(0.85)	6.52(0.86)
11	E_DIAG_	2010	S_FAM*1*_	P_FAM*1*_	***Main***	8.21(0.55)	7.88(0.57)	8.09(0.77)	7.66(0.85)	7.94(1.00)	7.45(1.15)
12	E_DIAG_	2011	S_FAM*1*_	P_FAM*1*_	***Main***	6.89(0.40)	6.74(0.40)	6.78(0.57)	6.61(0.59)	6.66(0.81)	6.50(0.85)

GEBV, genomic estimated breeding value; GV, genotypic value; SD=standard deviation.

a The mean square error value is from a linear model for the validation set, in which the GEBV is the covariate and the GV the response. The mean square error shown is the average of the mean square error over the different number of iterations (Table 2). Lower mean square error indicates more accurate and precise estimates of GEBV.

b GV are calculated using a phenotypic model with all of the lines.

c Marker effects from DIAG form are in bold with year of trial shown, and are equivalent to results from a single trial year analysis, marker effects for the MET analyses are in bold and italic, three marker effects are possible: main, interaction 2010, interaction 2011, with the sum of the (main + interaction) marker effects being equivalent of a total marker effect for a particular year.

In all three different CV evaluations [CV10, CV20, and CV40 (Table 3)], the *partitioned* model showed a higher R-squared and lower MSE than the *standard* model, indicating that the predictive ability and accuracy of line estimates (GEBVs) were superior in the *partitioned* model. This supports the finding of lower log-likelihoods and superior fit of the *partitioned* models as compared to the *standard* models when the full data were used (Table 4). The R-squared decreased, and the MSE increased, as the number of lines in the training set decreases (going from CV10 to CV40) for the equivalent model (*i.e.*, within the *partitioned* models, or within the *standard* models), which was expected, as predictions were based on fewer lines. It should be noted that, except where the 2011 marker effects from the P_DIAG_ model were used to generate the GEBVs (Comparisons 2 and 3, Table 6), the R-squared in the *partitioned* model in CV40 (where 40% of the lines were in the validation set) was similar to, or higher than, the R-squared of the *standard* model in CV10 (where 10% of the lines are in the validation set), suggesting the *partitioned* model was superior even when reducing the number of lines upon which the predictions are based. There was only a small compensating increase in MSE between the *standard* model in CV10, and the *partitioned* model in CV40, with the same exception of the 2011 marker effects from the P_DIAG_ model (Comparisons 2 and 3, Table 7), and also the main marker effects from the P_FAM_*_1_*, which showed larger increases (Comparison 12, Table 7). These results shows that the *partitioned* model provides the best predictions of the height of lines in the validation set.

The results of the CV10 cross-validation were considered in more detail, bearing in mind that the other cross-validations (CV20 and CV40) reflect similar patterns. Initially, results where the equivalent of a single trial analyses was used to generate both GEBVs (P_DIAG_ and S_DIAG_) and GVs (E_DIAG_) were examined. Using the same trial year to generate both the GEBVs and GVs (Comparisons 1 and 3, Table 6) gave the best results. The single trial analysis from 2010 showed a higher predictive ability than that from 2011 for the *partitioned* model (0.461 *vs.* 0.334, Table 6), and for the *standard* model (0.404 *vs.* 0.323, Table 6). This difference between the predictive ability in each year was initially surprising; given that there was a large overlap of individuals, the percentage of variation explained by the markers was similar, and the observations were highly correlated across the 2 years. The *partitioned* model showed a 5.7% and 1.1% improvement for 2010 and 2011, respectively, in predictive ability over the *standard* model.

If the opposite trial years were used to generate the GEBVs, then predicting 2010 GVs using the marker effects generated in 2011 (Comparisons 2, Table 6) had a higher predictive ability than the opposite combination (Comparisons 4, Table 6), for both the *partitioned* and *standard* models. Ly *et al.* (2013) suggested that G × E, which cannot be accounted for in a single trial, reduces the ability to make predictions. These results suggest that the 2011 heights had a higher environmental component than those observed in 2010, making prediction of the GEBVs from 2011 more difficult. We would therefore expect to be able to predict trial year 2011 better if a marker by trial interaction effect was included in a MET model, and this is exactly what was found, as discussed below.

The next comparisons are the MET analyses, where the GEBVs and GVs are based on a total effect found by summing the main or overall marker effect and the trial year marker effects. The first thing to note is that for 2010 the CS+DIAG form for $u_{\text{m}}$ had around a 4–6% higher predictive ability than the FAM*1* form in both the *standard* and *partitioned* models (Comparisons 5 and 7, Table 6). For 2011, the results were similar between the forms of $u_{\text{m}}$ (Comparisons 6 and 8, Table 6), with around a 0.1% difference in predictive ability between the FAM*1* form and the CS+DIAG form, with the latter showing only a slightly lower predictive ability, but a lower MSE. This suggests that the CS+DIAG form was superior to the FAM*1* form for this data set. Exploring different forms for $u_{\text{m}}$ is clearly an important step in determining the best model for GS.

The results for predictive ability were similar between the MET analysis and single year analysis for 2010 (Comparisons 5 and 1, 0.462 v 0.461, *partitioned* model, Table 6), but the MET shows a lower MSE (Comparisons 5 and 1, 7.35 v 7.47, *partitioned* model, Table 7). For 2011, the results of the MET were clearly superior over the single year analysis (Comparisons 6 and 3, Table 6), at 11.4% higher (0.448 v 0.334) for the *partitioned* model and 7.2% (0.395 v 0.323) for the *standard* model. This suggests that using 2 years’ data greatly improved the accuracy of the GEBVs by up to 11.4% for this data set. The benefit of including the marker by trial interaction effect was apparent in 2011 in particular, the year in which the results from the single trial analysis suggested that the environment had a large influence on the results. The greater environmental influence in 2011 is consistent with the lower heritability and reduced genetic influence on plant height compared to the 2010 trial (see previous section). Greater environmental stress may also explain the lower mean height of lines in 2011.

Finally, we compared the GEBVS derived from the main marker effects in a MET analysis with the GVs derived from the single year analysis. Again the CS+DIAG form was superior to the FAM*1* form for $u_{\text{m}}$ in terms of predictive ability, and we therefore concentrated on the former (Comparisons 9 and 10, Table 6). There was a reduction in predictive ability if the year specific marker effect was omitted when calculating the GEBVs, particularly for 2011 (0.448 *vs.* 0.336, Comparison 6 and 10, Table 6), where the environmental influence was higher. However, despite this reduction, a similar predictive ability to a single trial analysis was maintained, and the main effect still had a higher predictive ability than using the marker effects from the opposite year. For example, it is 3.5% higher in 2010 (Comparisons 9 and 2, 0.442 *vs.* 0.407), and 1.7% higher in 2011 (Comparisons 10 and 3, 0.336 *vs.* 0.319), with correspondingly lower MSE.

### Implementing GS with a lower density of markers

**Figure 2:**
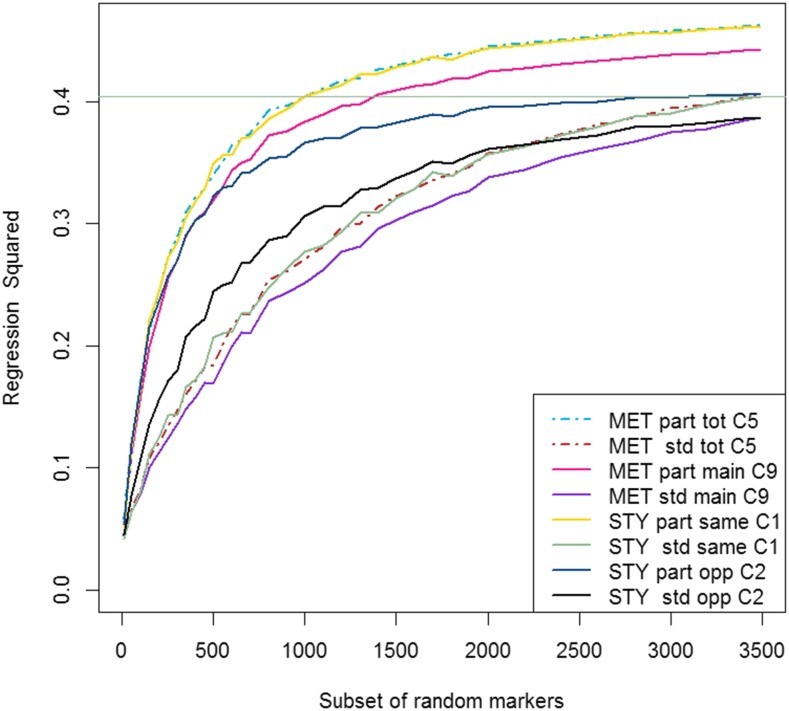
Comparison of *partitioned vs. standard* RR-BLUP model of CV10 (Table 3) for different forms (Table 1) and comparisons (Table 6) for trial year 2010 across a range of subsets of random markers. The horizontal line is maximum predictive ability of the *standard* single trial year analysis for 2010. Each subset represents the average results from 200 different sets of random markers, the comparisons across analyses are on the same subsets of random markers. MET, multi-environment trial analysis; STY, single trial year analysis; part, *partitioned* model; std, *standard* model; total, main marker effect + marker by trial interaction effect; main, main marker effect; same, same year used for prediction (2010 GEBV used to predict 2010 GV); opp, opposite year used for prediction (2011 GEBV used to predict 2010 GV); C, see Comparison as per Table 6 for more detail.

**Figure 3:**
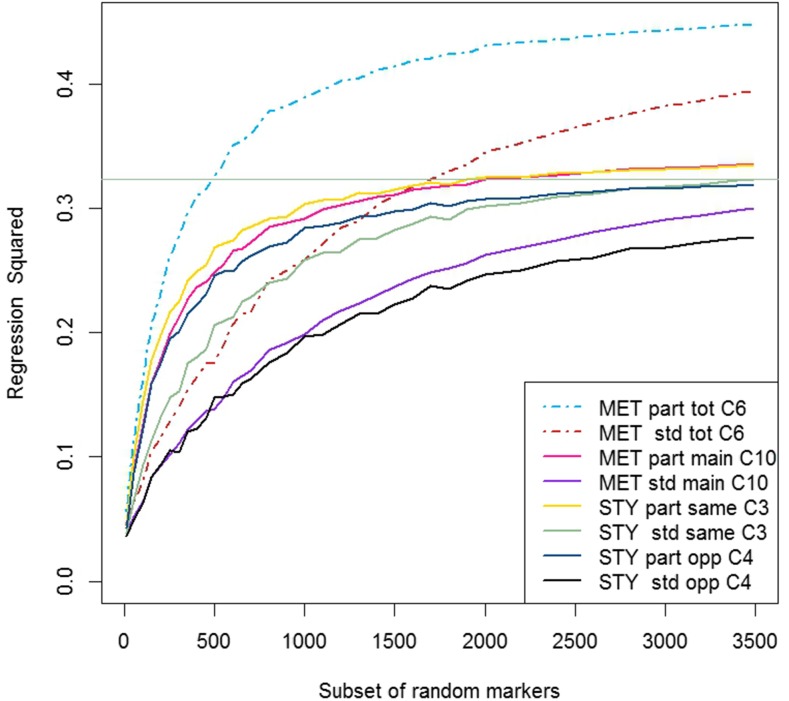
Comparison of *partitioned* verses *standard* RR-BLUP model of CV10 (Table 3) for different forms (Table 1) and comparisons (Table 6), for trial year 2011 across a range of subsets of random markers. The horizontal line is maximum predictive ability of the *standard* single trial year analysis for 2011. Each subset represents the average results from 200 different sets of random markers, the comparisons across analyses are on the same subsets of random markers. MET, multi-environment trial analysis; STY, single trial year analysis; part, *partitioned* model; std, *standard* model; total, main marker effect + marker by trial interaction effect; main, main marker effect; same, same year used for prediction (2011 GEBV used to predict 2011 GV); opp, opposite year used for prediction (2010 GEBV used to predict 2011 GV); C, see Comparison as per Table 6 for more detail.

A low density GS approach was considered, where, for each of the training sets of lines in CV10, the predictive ability of subsets of random markers was investigated. The results of the MET analyses and single trial analyses across the subsets of random markers were compared in each trial year (Figure 2 and Figure 3 for trial 2010 and trial 2011, respectively). Both plots showed that the *partitioned* models have a much steeper incline within the subsets of random markers containing less than 500 markers than the *standard* models, with the graphs flattening out more than the *standard* models as the marker numbers in subsets increase. This suggests that, for lower numbers of markers, the predictions were more accurate and reliable in the *partitioned* model, and therefore fewer markers were required to obtain similar predictions. This would tend to support the finding that the line estimates based on markers from the *partitioned* model were more accurate (lower MSE, Table 7) than those of the *standard* model. The horizontal lines in Figure 2 and Figure 3 show the single trial year analysis of the *standard* model using data from the same year. For trial 2010, all of the *partitioned* models had superior predictive ability across the entire range of marker subsets (Figure 2), with the main improvements in predictive ability coming within the first 1000 or so markers. In the *standard* RR-BLUP model, although there was an initial improvement in the predictive ability, this was less intense, and mostly small and steady over all of the latter marker subsets. This means that we can achieve the same predictive ability with the MET model as with the *standard* RR-BLUP model using around 1000 markers, or around 2490 markers less than in the full higher-density GS. In 2011, the *partitioned* models had superior performance over the *standard* single year model for lower numbers of markers, with the *partitioned* MET model reaching the same level of prediction with only 500 markers.

## Discussion

In this paper, a method for genomic selection is proposed for the analysis of multi-environment crop trials. The method differs from other methods in a number of ways.

The method uses raw data observations at the plot, or pot, level rather than line means, thus incorporating line replication. This enables the total genetic variation due to lines to be partitioned into variation due to markers, and residual genetic variation, where the latter accounts for any genetic variation unexplained by the markers. The GEBVs, which are based on marker effects, are assumed mostly to reflect additive effects. The residual genetic effect therefore should capture nonadditive effects. In inbred lines, the nonadditive effect will represent epistatic effects. However, in noninbred lines, other nonadditive effects will include dominance, inbreeding depression and homozygous dominance effects, the covariance between additive and dominance effects, and epistatic effects (de Boer and Hoeschele 1993).

Previous studies (Solberg *et al.* 2009; Crossa *et al.* 2010; Burgueno *et al.* 2012) have included pedigree information, which captures a polygenic effect as a way of accounting for nonadditive effects, and more recent studies (Da *et al.* 2014; Munoz *et al.* 2014) have used marker-based relationships to separate additive and nonadditive variation. Da *et al.* (2014) only included additive and dominance relationship matrices. Munoz *et al.* (2014) included, in addition, first-order epistatic relationship matrices (additive by additive, dominance by dominance, and additive by dominance); however, their approach has the disadvantage that the resulting genetic effects are nonorthogonal, and there is dependency between some of the estimates of the additive and nonadditive variance components, as the first-order epistatic relationship matrices they form are based on Hadamard products of the additive and dominance relationship matrices. This means that the matrices may not be capturing all nonadditive variation. Also, given the number of relationship matrices necessary to account for nonadditive variation, extension to a MET model may be difficult. In contrast, the residual genetic effect representing nonadditive effects found in our model is more encompassing and less restrictive, and should capture all nonadditive effects. It is, however, worth noting that, because of the flexibility of the linear mixed model, additional relationships matrices may also be added to our model if required. For example, if pedigree information was available, a single polygenic effect could be added, or it may be possible to further partition our residual genetic effects by the addition of further genomic relationship matrices, for instance, a genomic dominance matrix could be added in the case of hybrid crops.

Recent studies have shown that maximum prediction was reached when the breeding value was based on both additive and nonadditive effects (Da *et al.* 2014; Munoz *et al.* 2014), and Ly *et al.* (2013) notes that considering only the additive component may underestimate prediction accuracy. Here, we have only investigated the use of the additive proportion of the total genetic effect as described by the markers for the prediction of breeding values where, for future lines, *only* marker information is available. We found that partitioning the total genetic variation into marker and residual genetic variation, and using the improved predictive ability of the marker additive genetic effects for future predictions, gave more accurate estimates even for the single trial analysis than was the case if the total genetic variation was not partitioned (*i.e.*, when fitting the *standard* model, which excluded the residual genetic and therefore nonadditive, variation). Improvements of up to 5.7% in predictive ability were found. Further work is required to determine whether improved prediction can be achieved by using the total genetic effect (as opposed to the total genetic effect from markers) of a line from the *partitioned* model for phenotypic evaluation. The value of using the total genetic effect (additive plus nonadditive) for phenotypic evaluation would depend on the impact of the nonadditive proportion. If the nonadditive proportion of variation was reasonably high, the use of the total genetic effect for phenotypic evaluation would be important. However, it is worth noting the total genetic effect does not reflect the potential of a line as a parent as nonadditive effects are not inherited, although it may better predict the commercial viability of a line and may be useful in determining which lines to take forward from a breeding program for elite development.

Using the raw data in a single stage approach has the added advantage that it allows spatial variation to be incorporated into the analysis, enabling joint estimation of all effects, genetic and nongenetic. This is the preferred option, as the precorrection of data necessary in a two stage approach can have undesirable consequences, such as biased estimates of marker effects, and induced correlations between residuals (de los Campos *et al.* 2013).

It was evident from the analysis that the *standard* model had an inflated trial residual (error) term compared to the *partitioned* model. The *partitioned* model enabled the additive variation due to markers to be estimated, and it was found to account for approximately 75% of the total genetic variation in each of the trials. In the *standard* model, the inflation of the trial residual term, while apparent, was not sufficient to explain all of the unaccounted nonadditive genetic variation. This suggests that, in the *standard* model, some of the omitted nonadditive genetic variation is incorporated into the estimate of the genetic variation due to the markers, and they do not therefore reflect purely additive variation. This perhaps explains why, in the *standard* model, the marker effects are not as good for prediction of GEBVs, and this model showed higher MSE of prediction. The outcome is that estimates of marker effects from the *partitioned* model should be less biased, be more likely to reflect additive variation, and therefore lead to better estimation for future prediction.

The single trial RR-BLUP *partitioned* model was extended to enable a multi-environment approach to analysis. Analyzing trials under the *partitioned* RR-BLUP model in a MET setting extends a phenotypic MET model in an intuitive manner. In the GS model, we implicitly included both a main effect for markers, and a marker by trial interaction term. We found that a main marker effect in an analysis of trials in a MET was a useful predictor, even when a strong marker by trial interaction was present. These main marker effects seem to reflect a more ‘stable’ proportion of the marker, and were shown to have a predictive ability slightly superior to a single trial analysis. Presumably, the addition of more trials would improve the robustness of the main marker effect to G × E.

The RR-MET (*partitioned* and *standard*) performed well particularly where there was a larger environmental influence. In the example data set, in the 2011 trial, improvements of the MET over the single trial analysis of as much as 11.4% were seen, probably due to the inability of a single year analysis to account for the marker by trial variation. The poorer performance found when using 2011 marker effects to predict 2010 supports the observation of Ly *et al.* (2013) that the presence of G × E reduces the ability to make predictions in locations where no evaluations have previously been done. MET analysis uses the correlation between trials to improve prediction of lines (Smith *et al.* 2005). Where the environmental influence was lower, as in the 2010 trial, the *partitioned* single trial model performed well, with similar predictive ability to the *partitioned* MET model, but with lower MSE in the latter. As in Guo *et al.* (2013), gains here are attributable to the more accurate estimates of trial-specific marker effects through utilizing genetic correlations. In our cross-validation approach, we excluded validation lines from both trials, and found that, in 2011, where environmental influence was large, the MET was superior to the single trial analysis. Burgueno *et al.* (2012) and Guo *et al.* (2013) also looked at MET verses single trial analysis and included cross-validation schemes (referred to as CV1 in both papers), which also excluded validation lines from all environments. Our findings are contrary to those of Burgueno *et al.* (2012), who found no improvement in predictability of a MET over a single trial analysis, but support the findings of Guo *et al.* (2013), who found similar average gains in prediction accuracies of up to 10%.

In terms of using a subset of the markers in a low density GS approach, similar predictive ability could be gained with a much lower number of markers in the *partitioned* MET model, particularly in 2011. The results suggest that the *partitioned* model increased the accuracy of the marker effects with further gains to be had by using the MET model, particularly if the environmental influence on results is high (as in 2011). When examining the value of predictive ability of random subsets of markers, some subsets were superior to others (results not shown). Examining markers that are consistently found in the highly predictive subsets may lead to suitable choices for a MARS approach, and this may be worth exploring when considering the practical and optimal use of markers in GS in crops. Finally, as Heffner *et al.* (2011a, 2011b) have found, reducing the number of lines in a training population decreases the predictive ability. However, our observations suggest that the *partitioned* model goes some way to alleviating this effect.

In summary, the *partitioned* MET model used here is a single linear mixed model that incorporates trial, residual genetic-by-trial interactions, and trial-specific field and randomization-based terms, in a random RR-BLUP setting for marker effects and their interaction with trials. The MET model offers a viable, flexible addition to the GS tool box.

## Supplementary Material

Supplemental Material
