# Supplementary material for: Genomic Selection in Multi-environment Crop Trials
Source: G3 (Bethesda). 2016 Mar 11;6(5):1313–26. doi: 10.1534/g3.116.027524 (PMC4856083; doi:10.1534/g3.116.027524)
Supplement: Supplemental Material [file supp_6_5_1313__index.html]

Genomic Selection in Multi-environment Crop Trials — Supplemental Material 

# Genomic Selection in Multi-environment Crop Trials

## Supplemental Material for Oakey *et al.*, 2016

**Files in this Data Supplement:**

- File S1 - Instructions for analysis. Legends for File S2-S19. (.pdf, 266 KB)
- File S2 - 'datasetsGenetics.RData' contains the phenotypic and genotypic data in R data format. (.zip, 956 KB)
- File S3 - 'cv10sampling.R' is the R script for loading data set, the random division of the data and group combinations covering the 10 iterations for CV10. (.zip, 4 KB
- File S4 - 'cv20sampling.R' is the R script for loading data set, the random division of the data and group combinations covering the 45 iterations for CV20. (.zip, 4 KB)
- File S5 - 'cv40sampling.R' is the R script for loading data set, the random division of the data and group combinations covering the 210 iterations for CV40. (.zip, 5 KB)
- File S6 - Main R scripts for conducting the cross validation with each of the analysis. The scripts are customised for CV10. For CV20 and CV40 necessary amendments are documented in S1. 'single site - 2010.R' is the R script to produce results of comparison 1 (Table 6 and Table 7). (.zip, 3 KB)
- File S7 - Main R scripts for conducting the cross validation with each of the analysis. The scripts are customised for CV10. For CV20 and CV40 necessary amendments are documented in S1. 'single site - 2011.R' is the R script to produce results of comparison 3 (Table 6 and Table 7). (.zip, 3 KB)
- File S8 - Main R scripts for conducting the cross validation with each of the analysis. The scripts are customised for CV10. For CV20 and CV40 necessary amendments are documented in S1. 'single site - T10P11.R' is the R script to produce results of comparison 4 (Table 6 and Table 7). (.zip, 3 KB)
- File S9 - Main R scripts for conducting the cross validation with each of the analysis. The scripts are customised for CV10. For CV20 and CV40 necessary amendments are documented in S1. 'single site - T11P10.R' is the R script to produce results of comparison 2 (Table 6 and Table 7). (.zip, 3 KB)
- File S10 - Main R scripts for conducting the cross validation with each of the analysis. The scripts are customised for CV10. For CV20 and CV40 necessary amendments are documented in S1. 'met model CS+DIAG.R' is the R script to produce results of comparison 5, 6, 9, 10 (Table 6 and Table 7). (.zip, 3 KB)
- File S11 - Main R scripts for conducting the cross validation with each of the analysis. The scripts are customised for CV10. For CV20 and CV40 necessary amendments are documented in S1. 'met model -FAM1.R' is the R script to produce results of comparison 7, 8, 11, 12 (Table 6 and Table 7). (.zip, 4 KB)
- File S12 - R code for the MAS approach where a subset of random markers and their effects were used to predict GEBV. This was performed for CV10 only. 'calculate genetic predictions-subset markers AT random single site - 2010.R' is the R script for the MAS approach to support the results of comparison 1 (Table 6 and 7). (.zip, 2 KB)
- File S13 - R code for the MAS approach where a subset of random markers and their effects were used to predict GEBV. This was performed for CV10 only. 'calculate genetic predictions-subset markers AT random single site - 2011.R' is the R script for the MAS approach to support the results of comparison 3 (Table 6 and 7). (.zip, 2 KB)
- File S14 - R code for the MAS approach where a subset of random markers and their effects were used to predict GEBV. This was performed for CV10 only. 'calculate genetic predictions-subset markers AT random single site - T10P11.R' is the R script for the MAS approach to support the results of comparison 4 (Table 6 and 7). (.zip, 2 KB)
- File S15 - R code for the MAS approach where a subset of random markers and their effects were used to predict GEBV. This was performed for CV10 only. 'calculate genetic predictions-subset markers AT random single site - T11P10.R' is the R script for the MAS approach to support the results of comparison 2 (Table 6 and 7). (.zip, 2 KB)
- File S16 - R code for the MAS approach where a subset of random markers and their effects were used to predict GEBV. This was performed for CV10 only. 'calculate genetic predictions-subset markers AT random CS+DIAG.R' is the R script for the MAS approach to support the results of comparison 5, 6, 9, 10 (Table 6 and 7). (.zip, 3 KB)
- File S17 - R code for the MAS approach where a subset of random markers and their effects were used to predict GEBV. This was performed for CV10 only. 'calculate genetic predictions-subset markers AT random FAM1.R' is the R script for the MAS approach to support the results of comparison 7, 8, 11, 12 (Table 6 and 7). (.zip, 3 KB)
- File S18 - 'Figures 2 and 3.R' is the R script for producing Figure 2 and 3. (.zip, 2 KB)
- File S19 - 'Figure 1 Tables 4 and 5.R' is the R script for producing Figure 1 and results in Tables 4 and 5, the heritability and the proportion of variation accounted for by the markers. (.zip, 8 KB)
