# Supplementary material for: Genomic Selection in Multi-environment Crop Trials
Source: G3 (Bethesda). 2016 Mar 11;6(5):1313–26. doi: 10.1534/g3.116.027524 (PMC4856083; doi:10.1534/g3.116.027524)
Supplement: Supplemental Material [file supp_g3.116.027524_FileS1.pdf]

File S1

Instructions for analysis

Analysis was performed using R v3.2.0 (R Core Team 2015) using the library ASReml v3.0-1 (Butler *et al.* 2009). To reproduce the analysis using the code provided in the supplementary files the following set up of folders is suggested. It is suggested to create the following FOLDERS and subfolders placing the listed supplementary files in them.

DATA File S2 'datasetsGenetics.RData'

CV10

sampling File S3 'cv10sampling.R'
single site – 2010 File S6 'single site – 2010.R', File S12 'calculate genetic predictions-subset markers AT random single site – 2010.R'
single site – 2011 File S7 'single site – 2011.R', File S13 'calculate genetic predictions-subset markers AT random single site – 2011.R'
single site - T10P11 File S8 'single site – T10P11.R', File S14 'calculate genetic predictions-subset markers AT random single site – T10P11.R'
single site – T11P10 File S9 'single site – T11P10.R', File S15 'calculate genetic predictions-subset markers AT random single site – T11P10.R'
met model -CS +DIAG File S10 'met model CS+DIAG.R', File S16 'calculate genetic predictions-subset markers AT random CS+DIAG.R'
met model -FAM1 File S11 'met model -FAM1.R', File S17 'calculate genetic predictions-subset markers AT random FAM1.R'

For analysis start with running the scripts in the sampling subfolder, then proceed with other folders.

Folders CV20 and CV40 and the corresponding subfolders can also be created with the following differences

a. the R scripts copied to the 'sampling' subfolders will be File S4 and File S5 for CV20 and CV40 respectively and need no further alteration
b. the R script for Files S12-S17 are not required in CV20 and CV40
c. the other scripts Files S6-S11 that are supplied are set up to run CV10 and some minor modifications to Files S6-S11 of CV20 and CV40 are therefore necessary. The changes required are identified within the R scripts but are also documented below

Changes to the Files S6-S11 for CV20 and CV40

Within the SIM section of the R code

i. For CV20 replace s<-10 with s<-45
ii. For CV40 replace s<-10 with s<-210
iii. place # in front of (or) delete line
#eval(parse(file="calculate genetic predictions-subset markers AT random CS+DIAG.R"))

It is not necessary to run the code under the EXPORT section as this is used to create Figures 2 and 3 from CV10 results only

Files S2-S19

Available for download at www.g3journal.org/lookup/suppl/doi:10.1534/g3.116.027524 /-/DC1

File S2 ‘datasetsGenetics.RData’ contains the phenotypic and genotypic data in R data format

File S3 ‘cv10sampling.R’ is the R script for loading data set, the random division of the data and group combinations covering the 10 iterations for CV10

File S4 ‘cv20sampling.R’ is the R script for loading data set, the random division of the data and group combinations covering the 45 iterations for CV20

File S5 ‘cv40sampling.R’ is the R script for loading data set, the random division of the data and group combinations covering the 210 iterations for CV40

Files S6-S11: Main R scripts for conducting the cross validation with each of the analysis. The scripts are customised for CV10. For CV20 and CV40 necessary amendments are documented in S1

File S6 ‘single site – 2010.R’ is the R script to produce results of comparison 1 (Table 6 and Table 7)

File S7 ‘single site – 2011.R’ is the R script to produce results of comparison 3 (Table 6 and Table 7)

File S8 ‘single site – T10P11.R’ is the R script to produce results of comparison 4 (Table 6 and Table 7)

File S9 ‘single site – T11P10.R’ is the R script to produce results of comparison 2 (Table 6 and Table 7)

File S10 ‘met model CS+DIAG.R’ is the R script to produce results of comparison 5, 6, 9, 10 (Table 6 and Table 7)

File S11 ‘met model -FAM1.R’ is the R script to produce results of comparison 7, 8, 11, 12 (Table 6 and Table 7)

File S12-S17 R code for the MAS approach where a subset of random markers and their effects were used to predict GEBV. This was performed for CV10 only.

File S12 ‘calculate genetic predictions-subset markers AT random single site – 2010.R’ is the R script for the MAS approach to support the results of comparison 1 (Table 6 and 7)

File S13 ‘calculate genetic predictions-subset markers AT random single site – 2011.R’ is the R script for the MAS approach to support the results of comparison 3 (Table 6 and 7)

File S14 ‘calculate genetic predictions-subset markers AT random single site – T10P11.R’ is the R script for the MAS approach to support the results of comparison 4 (Table 6 and 7)

File S15 ‘calculate genetic predictions-subset markers AT random single site – T11P10.R’ is the R script for the MAS approach to support the results of comparison 2 (Table 6 and 7)

File S16 ‘calculate genetic predictions-subset markers AT random CS+DIAG.R’ is the R script for the MAS approach to support the results of comparison 5, 6, 9, 10 (Table 6 and 7)

File S17 ‘calculate genetic predictions-subset markers AT random FAM1.R’ is the R script for the MAS approach to support the results of comparison 7, 8, 11, 12 (Table 6 and 7)

File S18 ‘Figures 2 and 3.R’ is the R script for producing Figure 2 and 3

File S19 ‘Figure 1 Tables 4 and 5.R’ is the R script for producing Figure 1 and results in Tables 4 and 5, the heritability and the proportion of variation accounted for by the markers
